# Cranial irradiation alters neuroinflammation and neural proliferation in the pituitary gland and induces late‐onset hormone deficiency

**DOI:** 10.1111/jcmm.16086

**Published:** 2020-11-10

**Authors:** Yiran Xu, Yanyan Sun, Kai Zhou, Cuicui Xie, Tao Li, Yafeng Wang, Yaodong Zhang, Juan Rodriguez, Xiaoan Zhang, Ruijin Shao, Xiaoyang Wang, Changlian Zhu

**Affiliations:** ^1^ Henan Key Laboratory of Child Brain Injury Institute of Neuroscience and Third Affiliated Hospital of Zhengzhou University Zhengzhou China; ^2^ Center for Brain Repair and Rehabilitation Institute of Neuroscience and Physiology University of Gothenburg Gothenburg Sweden; ^3^ Department of Human Anatomy School of Basic Medical Sciences Zhengzhou University Henan China; ^4^ Department of Women’s and Children’s Health Karolinska Institute Stockholm Sweden; ^5^ Department of Neonatology Children’s Hospital of Zhengzhou University Zhengzhou China; ^6^ Department of Physiology/Endocrinology Sahlgrenska Academy at University of Gothenburg Gothenburg Sweden; ^7^ Perinatal Center Institute of Neuroscience and Physiology Sahlgrenska Academy University of Gothenburg Gothenburg Sweden

**Keywords:** cancer survivors, cranial irradiation, hypopituitarism, pituitary gland

## Abstract

Cranial radiotherapy induces endocrine disorders and reproductive abnormalities, particularly in long‐term female cancer survivors, and this might in part be caused by injury to the pituitary gland, but the underlying mechanisms are unknown. The aim of this study was to investigate the influence of cranial irradiation on the pituitary gland and related endocrine function. Female Wistar rat pups on postnatal day 11 were subjected to a single dose of 6 Gy whole‐head irradiation, and hormone levels and organ structure in the reproductive system were examined at 20 weeks after irradiation. We found that brain irradiation reduced cell proliferation and induced persistent inflammation in the pituitary gland. The whole transcriptome analysis of the pituitary gland revealed that apoptosis and inflammation‐related pathways were up‐regulated after irradiation. In addition, irradiation led to significantly decreased levels of the pituitary hormones, growth hormone, adrenocorticotropic hormone, thyroid‐stimulating hormone and the reproductive hormones testosterone and progesterone. To conclude, brain radiation induces reduction of pituitary and reproduction‐related hormone secretion, this may due to reduced cell proliferation and increased pituitary inflammation after irradiation. Our results thus provide additional insight into the molecular mechanisms underlying complications after head irradiation and contribute to the discovery of preventive and therapeutic strategies related to brain injury following irradiation.

## 1 INTRODUCTION

Almost one third of malignancies in children are brain tumours, and the incidence has increased in recent decades. Improved treatments have considerably increased survival, and today 40%‐80% of childhood brain tumour patients in most countries survive their disease.^1^ However, a recent study found that about half of childhood cancer survivors develop a severe or life‐threatening chronic health condition by the age of 50 years,^2^ and the tough treatment, particularly cranial radiotherapy (CRT), has both acute and long‐lasting side effects. The developing brain is particularly susceptible to the negative effects of CRT, resulting not only in intellectual impairment,^3^, ^4^, ^5^, ^6^, ^7^ but also in endocrine sequelae such as growth retardation and reproductive abnormalities.^8^ Radiotherapy‐induced endocrine and reproductive complications occur in patients with pituitary tumours, nasopharyngeal carcinoma, retinoblastoma, and other tumours in the hypothalamus‐pituitary region and in patients receiving whole‐brain irradiation. In addition, children who receive prophylactic whole‐brain irradiation for acute lymphoblastic leukaemia also have a high risk of endocrine problems after many years and are especially prone to insufficient growth hormone secretion.^9^, ^10^, ^11^, ^12^ Although there are many new methods (proton beam, Leksell gamma knife and stereotactic linear charger) to deliver radiation to tumours, some studies indicate that endocrine and reproductive complications still occur.^13^, ^14^ Radiotherapy‐induced endocrine and reproductive dysfunction have significant impacts on quality of life and have become an important issue in the follow‐up of patients who receive CRT in childhood.^15^, ^16^

The pituitary is a pea‐sized gland that sits in a protective bony enclosure called the sella turcica, and it is a complex area of the brain involved in endocrine function and reproduction regulation.^17^ Cranial irradiation has multiple side effects on the pituitary gland, including hypopituitarism (growth hormone, GH; adrenocorticotropic hormone, ACTH; and thyroid‐stimulating hormone, TSH; deficiencies), cerebrovascular morbidity, optic neuropathy and hypogonadism.^18^ Currently, there is no proven strategy to prevent radiotherapy‐induced pituitary dysfunction, and these irradiation‐induced hormone deficiencies and complications are irreversible and progressive.^19^, ^20^ Current research is focused primarily on the follow‐up of clinical patients, and there are few studies on the mechanism of pituitary injury after brain irradiation. The current therapy for pituitary injury is still mainly supportive, and thus there is an urgent and persistent need for a better understanding of the mechanisms of pituitary injury after brain irradiation. The purpose of this study was to define both short‐term and long‐term endocrine and reproductive complications and to use transcriptome analysis to identify potential mechanisms leading to the changes in the pituitary after cranial irradiation in juvenile rats.

## 2 MATERIAL AND METHODS

### 2.1 Animals and irradiation procedure

Female Wistar rat pups were purchased from Charles Rivers Laboratories (Sulzfeld, Germany) and were housed in pathogen‐free temperature and humidity‐controlled IVC cage (Tecniplast, Italy) at the Experimental Biomedical centre of the University of Gothenburg with a 12‐hour light‐dark cycle with food and water available ad libitum. The animal inclusion criteria for this study were rats with body weight 20.0‐30.0 g at postnatal day (P) 11 and each litter with 10 pups. A total of 40 rat pups were used for analysis in this study, and the pups were randomly assigned to each group. The rat pups were anaesthetized with a 50 mg/kg intraperitoneal injection of tribromoethanol (Avertin, Sigma‐Aldrich, Stockholm, Sweden) on P11 and placed in a prone position (head to gantry) on a Styrofoam bed. Each animal was given 6 Gy radiation, which is equivalent to a clinical dose of 12 Gy (repeated daily 2 Gy fractions), and this represents a clinically relevant low‐ and medium‐level irradiation.^21^ The detailed irradiation procedure was described previously.^22^ The controls were anaesthetized but not irradiated. All experiments were approved by the Gothenburg Committee of the Swedish Animal Welfare Agency (application no. 2014‐112) and followed the animal experiments guidelines of Gothenburg University.

### 2.2 Tissue preparation

The rats were deeply anaesthetized with pentobarbital sodium and then perfused with ice cold phosphate buffered saline (PBS) at 20 weeks after brain irradiation. After decapitation, the pituitary gland was quickly removed and frozen in dry ice and stored at −80°C. Ovarian and uterine tissue samples were fixed in 4% formaldehyde buffer for 24 hours, dehydrated with gradient ethanol and xylene, and embedded in paraffin.

### 2.3 Haematoxylin and eosin staining (H&E)

The paraffin sections of ovarian and uterine tissues and rat vaginal epithelial cell smears were stained with H&E.

### 2.4 Oestrous cycle detection

At 15 weeks after irradiation, vaginal smears were performed daily for 15 consecutive days to determine the periodicity of the oestrous cycle. According to the vaginal smear results, the animals were divided into dioestrus, pro‐oestrus, oestrus and metoestrus phases.

### 2.5 BrdU administration

To label proliferating cells before irradiation, freshly prepared thymine analog 5‐bromo‐2‐deoxyuridine (BrdU) (Roche, 5 mg/mL in 0.9% saline) was injected intraperitoneally at a dose of 50 mg/kg on P9 and P10.

### 2.6 Immunohistochemistry staining and cell counting

At 20 weeks after irradiation, the pituitary gland was removed by perfusion with PBS after anaesthetization, embedded in dehydrated paraffin and cut into 5 μm sagittal sections. The primary antibody was rat anti‐BrdU (1:100 dilution; clone: BU1/75, Nordic BioSite, Stockholm, Sweden), and the secondary antibody was goat anti‐rat (1:200 dilution; Vector Laboratories, Burlingame, CA, USA). The detailed procedure was described previously.^23^ The BrdU‐positive cells were counted at 200 × magnification in the pituitary gland area using a microscope, and the corresponding area of each region was measured using ImageJ 1.80 software (NIH, Bethesda, MD). Two sections were counted from each pituitary with an interval of 100 μm, and all of the counting was carried out by investigators blinded to group assignment.

### 2.7 RNA extraction and sequencing

At 6 hours after irradiation, 20 pituitary samples from irradiated and control rats were prepared for RNA sequencing. Total RNA was extracted from each sample using the RNeasy Mini kit (Qiagen, Hilden, Germany), and the library preparation was completed using the MGI Easy mRNA library preparation kit (BGI, Inc, Wuhan, China) according to the manufacturer's instructions.^22^ Using Rattus_norvegicus‐UCSC_rn6 as the reference genome (ftp://hgdownload.cse.ucsc.edu/goldenPath/rn6), and according to the criterion of adjusted *P* value < 0.05, the DESeq method was used to screen differentially expressed genes between the two groups. The transcriptome sequencing data generated in this study have been deposited in NCBI BioSampledatabase (https://www.ncbi.nlm.nih.gov/bioproject/PRJNA528821).

### 2.8 Pituitary and reproduction‐related hormones and multiplex cytokine/chemokine assay

Blood samples were collected from the tail in the morning around 10 am and placed at room temperature for about 60 minutes to coagulate and then centrifuged at 1000 × *g* for 10 minutes at 5 and 20 weeks after irradiation. The serum was then removed and stored at −80°C. A Luminex assay was used according to the manufacturer's instructions (Millipore, Stockholm, Sweden) to detect pituitary‐related hormones (MILLIPLEX MAP rat pituitary magnetic bead panel assay kit). Reproduction‐related hormone analyses were performed using ultrasensitive rat estradiol, testosterone and progesterone ELISA kits (Crystal Chem, IL, USA). To compare pro‐inflammatory mediator expression profiles between irradiated and non‐irradiated control rats, a Luminex assay was used according to the manufacturer's instructions (Millipore, Stockholm, Sweden). The results were analysed using a Bio‐Plex workstation (Bio‐Rad, Hercules, CA, USA) and normalized to the amount of total protein extracted from hypothalamus homogenates using a colorimetric Bio‐Rad DC Protein Assay kit (Bio‐Rad).

### 2.9 Statistical analysis

All analyses used the Statistical Product and Service Solutions 17.0 (SPSS, IBM, New York, USA). The comparison between the two groups was conducted by Student's *t* test, and the data with unequal variances were compared with the Mann‐Whitney *U* test. The results are expressed as mean ± SEM, and *P* < 0.05 is considered statistically significant.

## 3 RESULTS

### 3.1 Irradiation reduces the survival of proliferating cells in the pituitary gland

**Figure 1:**
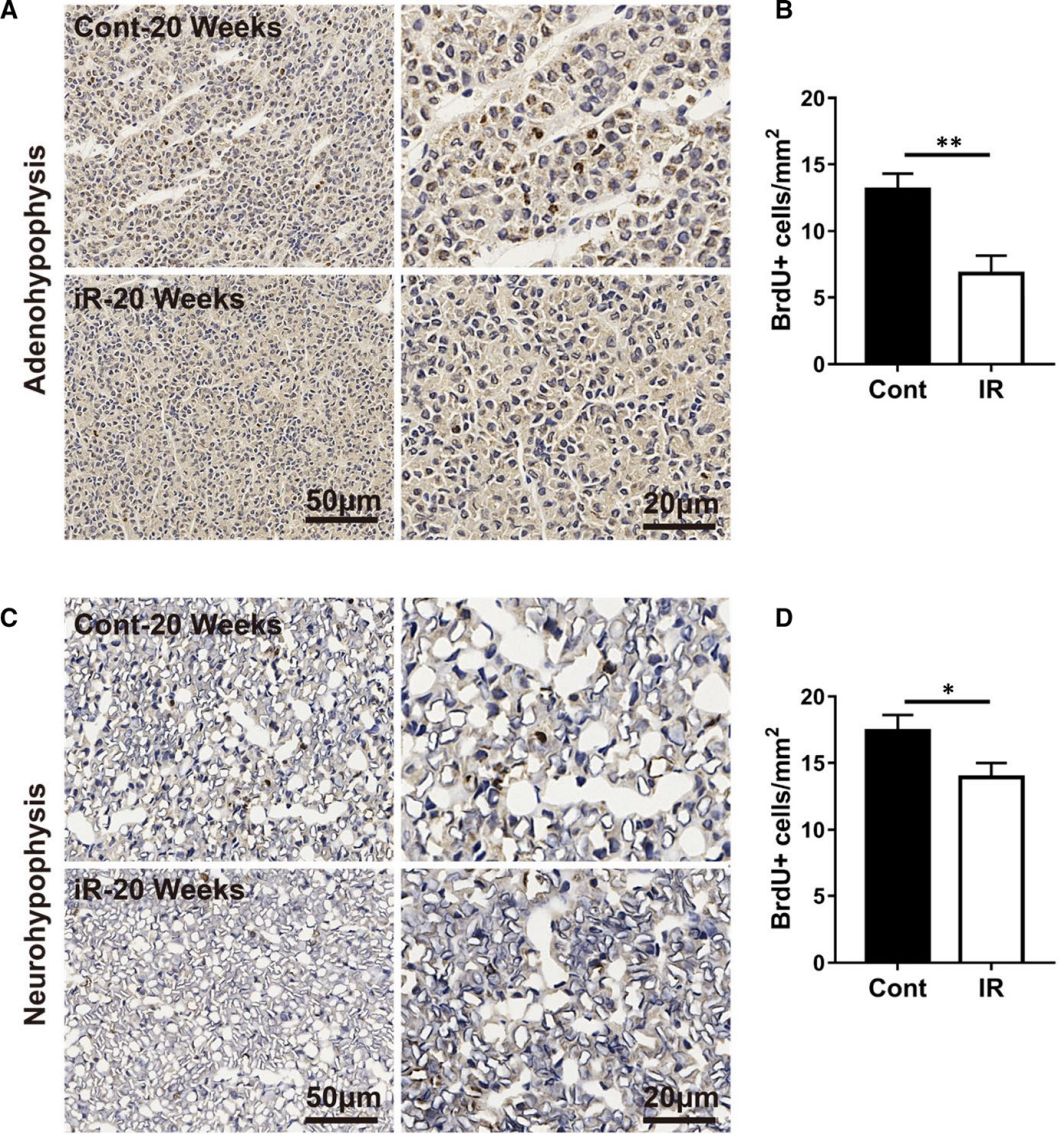
Irradiation reduces the survival of proliferating cells in the pituitary gland. A, Representative BrdU staining of the adenohypophysis. B, Quantification of BrdU‐labelled cells indicating the number of proliferating cells surviving at 20 wk. C. Representative BrdU staining of the neurohypophysis. D. Quantification of BrdU‐labelled cells. n = 10/group, *P < 0.05, **P < 0.01

To understand the effect of irradiation on cell proliferation, the survival of proliferating cells in the pituitary gland was measured by BrdU labelling immediately after irradiation followed by evaluation at 20 weeks. In both the adenohypophysis (*P* < 0.05) and neurohypophysis regions (*P* < 0.01), the number of BrdU‐labelled cells was significantly reduced in the irradiated group compared with the non‐irradiated group (Figure 1A‐D).

### 3.2 Irradiation induces transcriptome alterations in the pituitary gland

**Figure 2:**
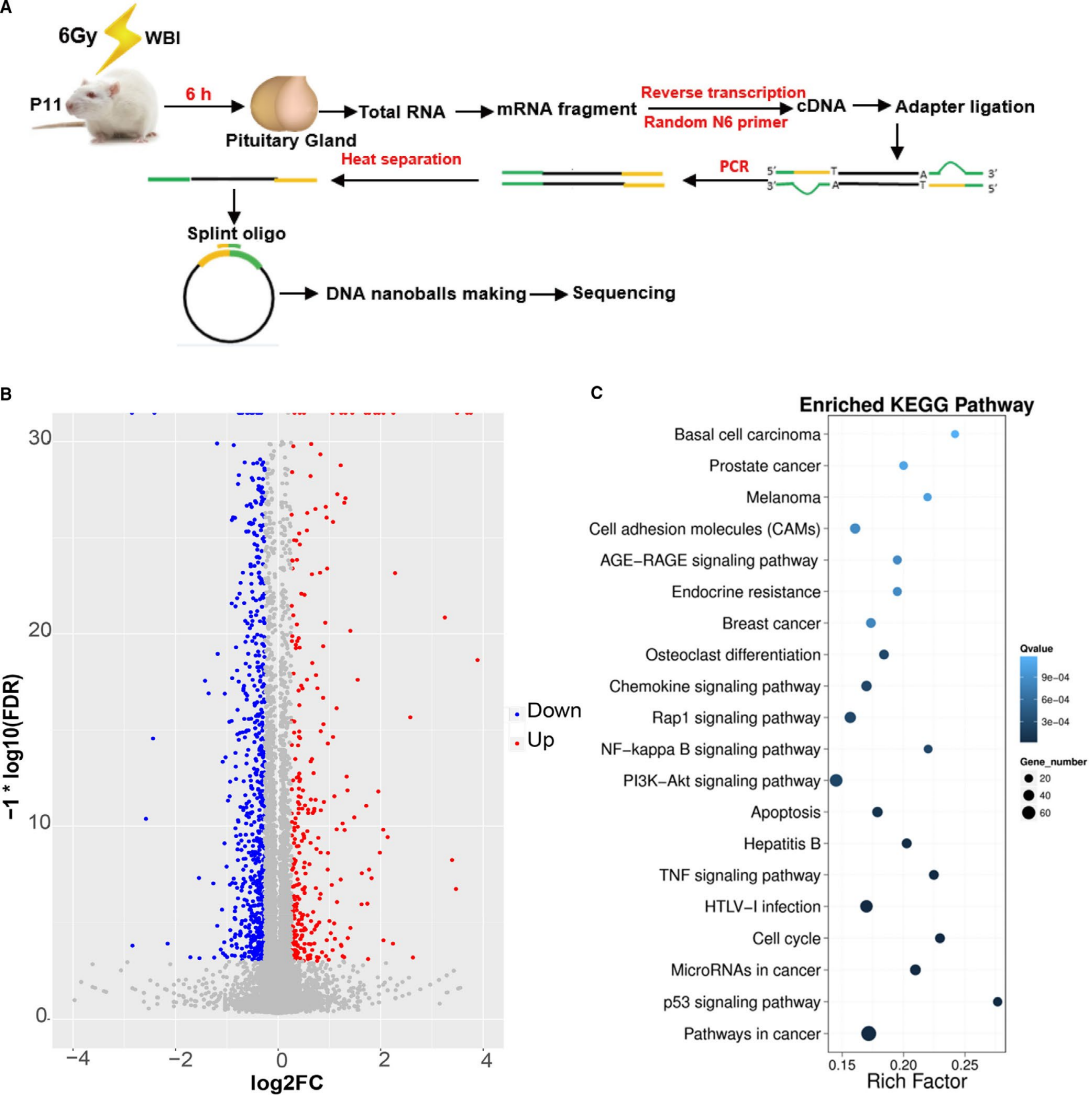
Irradiation induces transcriptome alterations in the pituitary gland. A, The processes for analysing the transcriptome. B, Scatter plots of all expressed genes in each pairwise RNA sequence analysis in the pituitary gland at 6 h after irradiation. C, KEGG pathway enrichment analyses of all significantly altered genes from the pituitary gland after irradiation. n = 10/group

To investigate the impact of cranial irradiation on mRNA expression in the pituitary gland, the transcriptome of the female rat pituitary gland was assayed by RNA sequencing at 6 hours after irradiation (Figure 2A). The data analysis showed that 1334 out of the total of 16 026 genes were significantly differentially expressed in the irradiated pituitary gland compared to non‐irradiated controls (Figure 2B), and of these genes 451 were up‐regulated and 883 were down‐regulated in the irradiation group. KEGG pathway enrichment analysis showed that genes involved in endocrine resistance, the inflammation‐related pathways (such as chemokine signalling pathway, NF‐kB signalling pathway, and TNF signalling pathway), apoptosis, and the p53 signalling pathway were significantly differentially expressed in the irradiation group (Figure 2C).

### 3.3 Irradiation induces persistent inflammation in the pituitary gland

**Figure 3:**
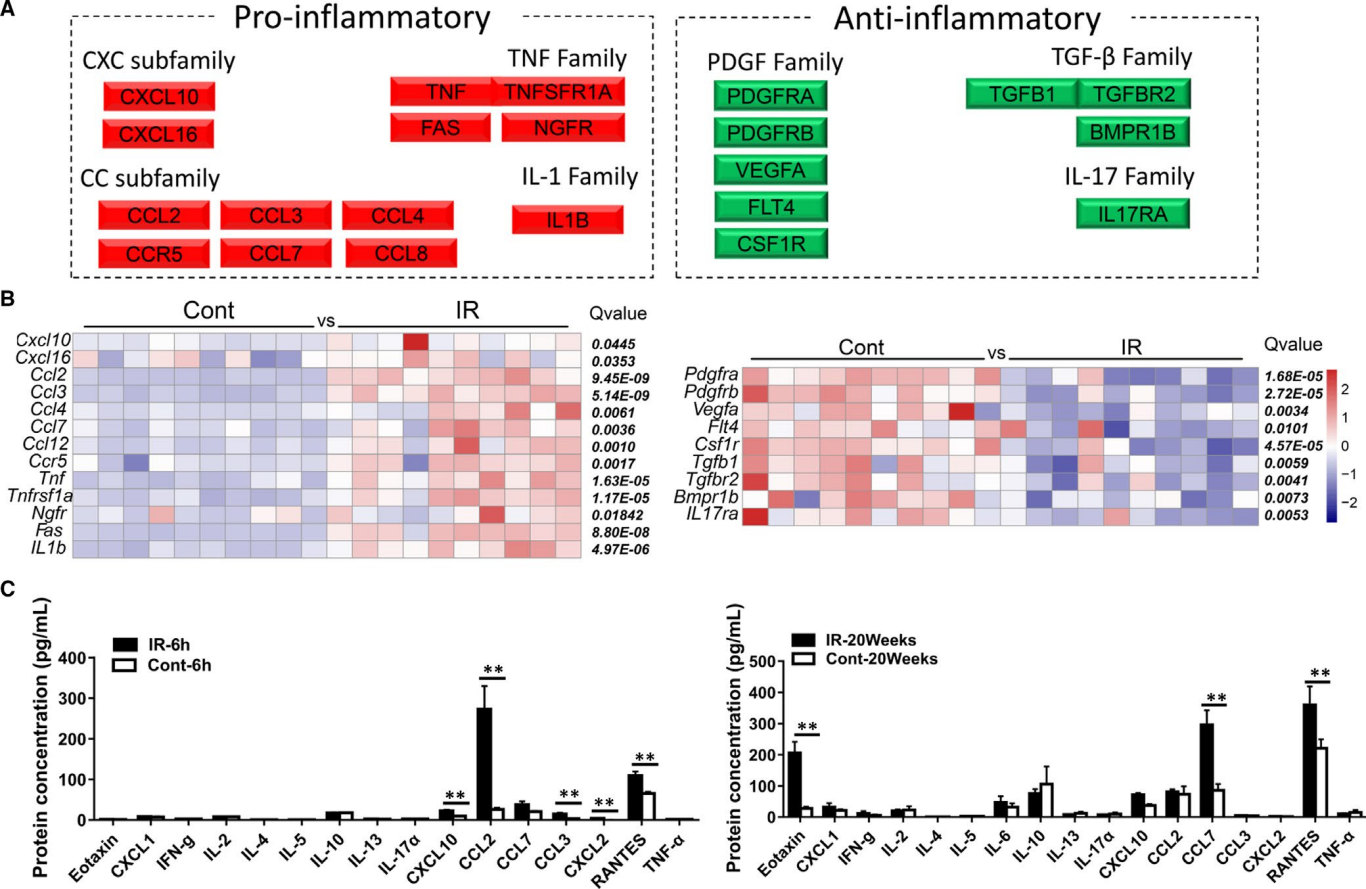
Irradiation induces persistent inflammation in the pituitary gland. A, The change of inflammation‐related genes in the pituitary gland. B, Heatmap of inflammation‐related DEGs. C, Luminex assay of cytokines/chemokines from the cytosolic fraction of the pituitary gland at 6 h and 20 wk after irradiation. n = 10/group, *P < 0.05, **P < 0.01, ***P < 0.001

Because we found that a large portion of the genes that were differently regulated in the pituitary gland after irradiation were inflammation‐related genes, we conducted further analysis of these genes and classified them into pro‐inflammatory and anti‐inflammatory genes according to their functions (Figure 3A). Among them, expression of the pro‐inflammatory genes *Cxcl10*, *Cxcl16*, *Ccl2*, *Ccl3*, *Ccl4*, *Ccl7*, *Ccl12*, *Ccr5*, *Tnf*, *Tnfrsf1a*, *Ngfr*, *Fas* and *IL1b* was significantly increased in the irradiation group compared with the non‐irradiation control group (the left panel in Figure 3B), while the anti‐inflammatory genes *Pdgfra*, *Pdgfrb*, *Vegfa*, *Flt4*, *Csf1r*, *Tgfb1*, *Tgfbr2*, *Bmpr1b* and *IL12ra* were significantly decreased in the irradiation group (the right panel in Figure 3B). To further confirm these findings, the protein level of cytokines and chemokines in the cytosolic fraction of the pituitary gland was measured at 6 hours and 20 weeks after irradiation with the Luminex assay (the left panel in Figure 3C). The protein expression of CXCL‐10, CCL2, CCL7, CCL3, CXCL2 and RANTES was increased significantly already as early as 6 hours after irradiation (*P* < 0.01). The irradiation‐induced inflammatory reaction was persistence, and the protein expression of Eotaxin, CXCL10 and CCL7 remained significantly higher in the irradiation group compared to controls at 20 weeks after irradiation (*P* < 0.01) (the right panel in Figure 3C). Furthermore, to better understand the changes in inflammation in the pituitary gland after irradiation, we analysed TNF signalling pathway‐related gene expression. Our results show that most genes were significantly up‐regulated (Figure S1A), including *TNF*, *TNFR1*, *CIAP1/2*, *NIK*, *CASP3*, *IκBα*, *C/EBPβ*, *P13K*, *Ccl12*, *Ccl2*, *Cxcl10*, *Csf1*, *Fas*, *IL1b*, *Lif* and *Icam1*, in the irradiated group compared with the non‐irradiated control group (Figure S1B).

### 3.4 Irradiation increased the expression level of genes involved in the p53 signalling pathway in the pituitary gland

**Figure 4:**
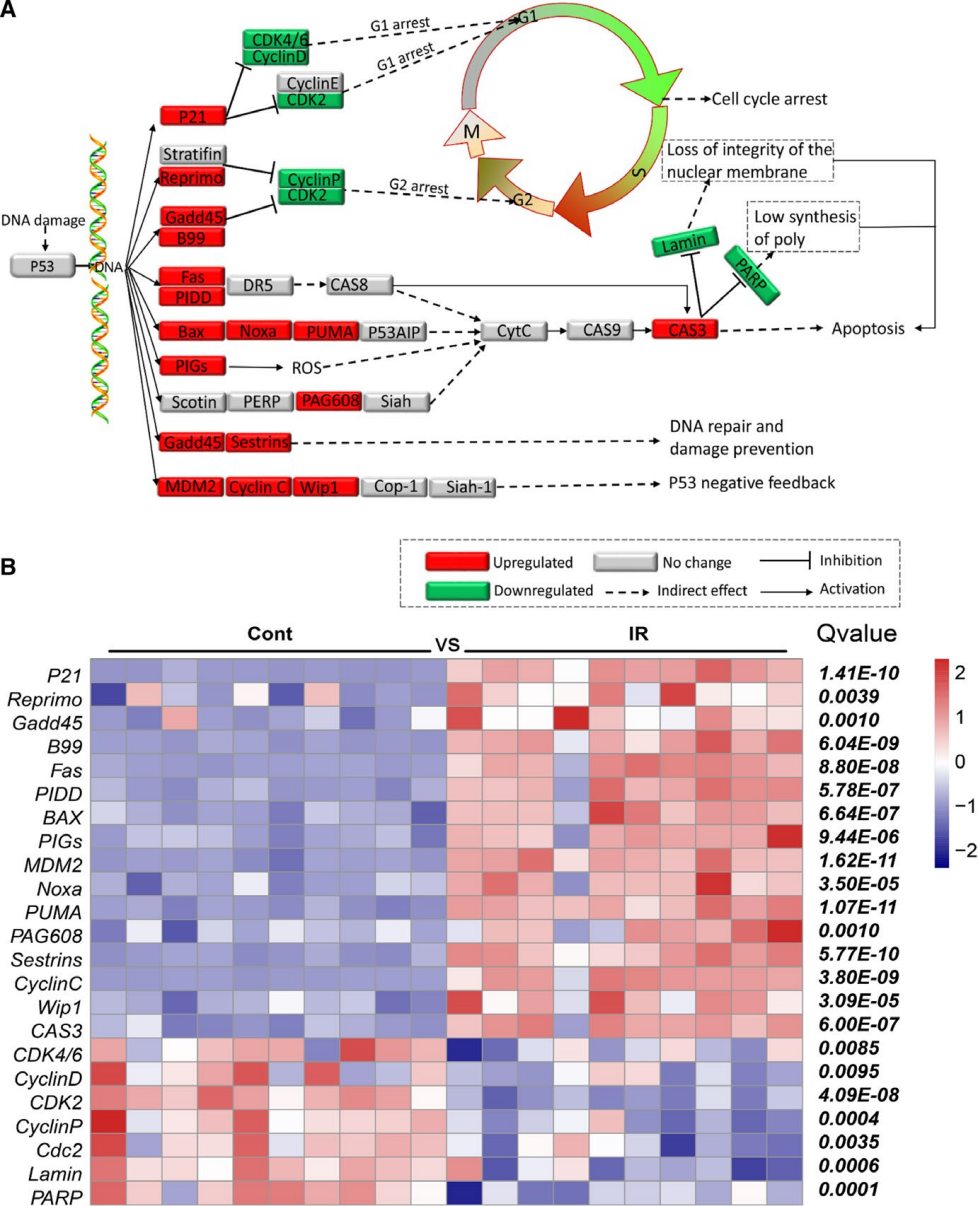
The expression of genes of the p53 signalling pathway in the pituitary gland. A, The pattern of p53 signalling pathway genes, with reduced expression shown in green and increased expression shown in red. B. Heatmap of p53 signalling pathway‐related DEGs

To understand the potential changes to the pituitary gland after brain irradiation, we used pathway enrichment to analyse the gene expression of the apoptosis related‐p53 signalling pathway (Figure 4A). Expression of the *B99*, *Fas*, *p21*, *Reprimo*, *Gadd45*, *PIDD*, *BAX*, *PIGs*, *MDM2*, *Noxa*, *PUMA*, *PAG608*, *Sestrins*, *CyclinC*, *Wip1* and *CAS3* genes was increased significantly in the irradiation group compared to non‐irradiated controls, and expression of *CDK4/6*, *CyclinD*, *CDK2*, *CyclinP*, *Cdc2*, *Lamin* and *PARP* was significantly decreased (Figure 4B). These results indicated that the p53 signalling pathway was activated in the pituitary after irradiation.

### 3.5 Irradiation decreased the expression of genes involved in the Hippo signalling pathway in the pituitary gland

**Figure 5:**
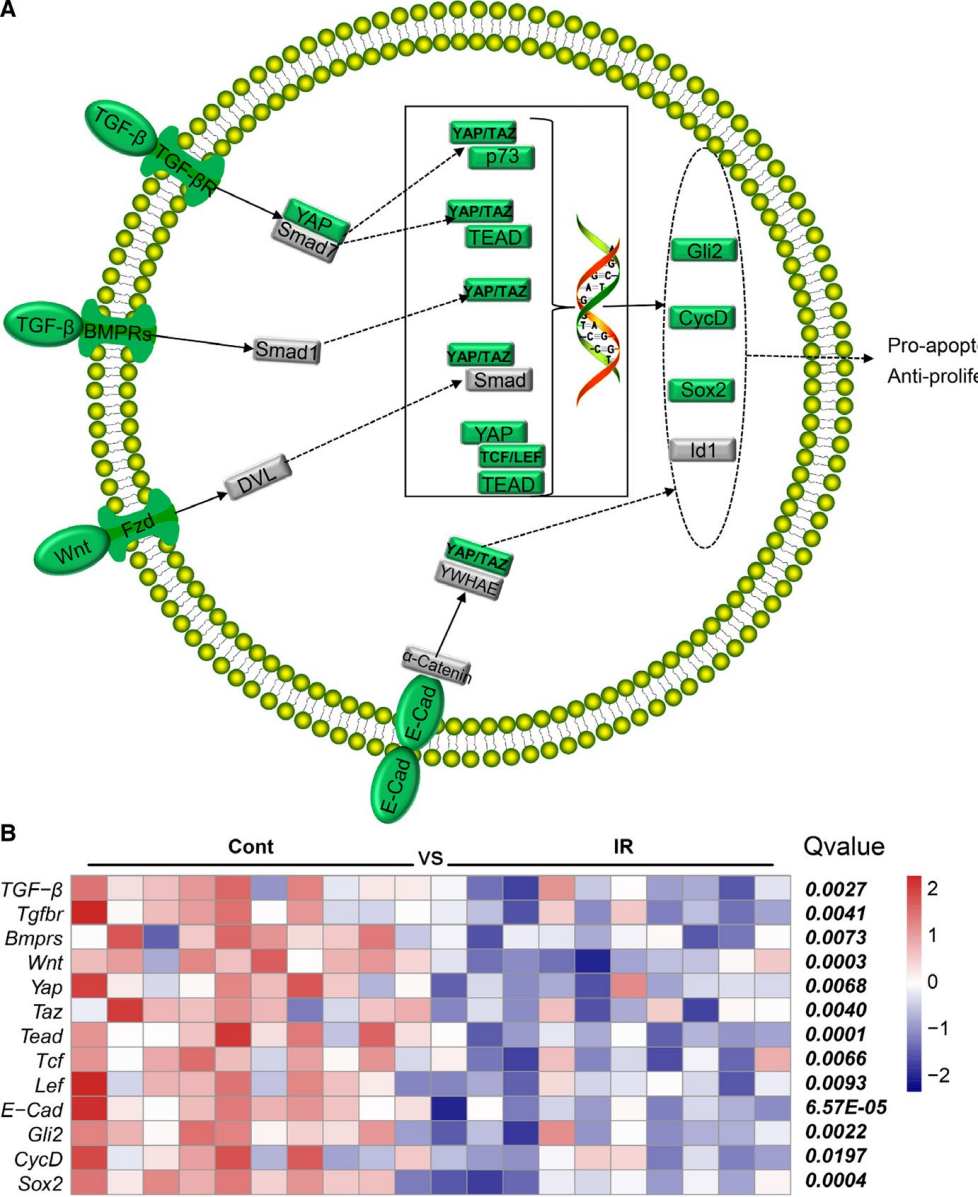
The expression of genes of the Hippo signalling pathway in the pituitary gland. A, The pattern of Hippo signalling pathway genes. B. Heatmap of Hippo signalling pathway‐related DEGs

To further analyse the apoptosis/cell proliferation‐related signalling pathways, we performed further analysis of the Hippo pathway using the obtained transcriptome data base. The Hippo signalling pathway plays an evolutionarily conserved role in organ size control by inhibiting cell proliferation, promoting apoptosis, regulating fates of stem cells, and limiting cell size under some circumstances (Figure 5A). We found that expression of the Hippo pathway‐related genes *TGF‐β*, *Tgfbr*, *Bmprs*, *Wnt*, *Yap*, *Taz*, *Tead*, *Tcf*, *Lef*, *E‐Cad*, *Gli2*, *CycD* and *Sox2* was significantly decreased after irradiation (Figure 5B).

### 3.6 Irradiation of the juvenile female rat brain induces adult pituitary‐related hormone deficiency

**Figure 6:**
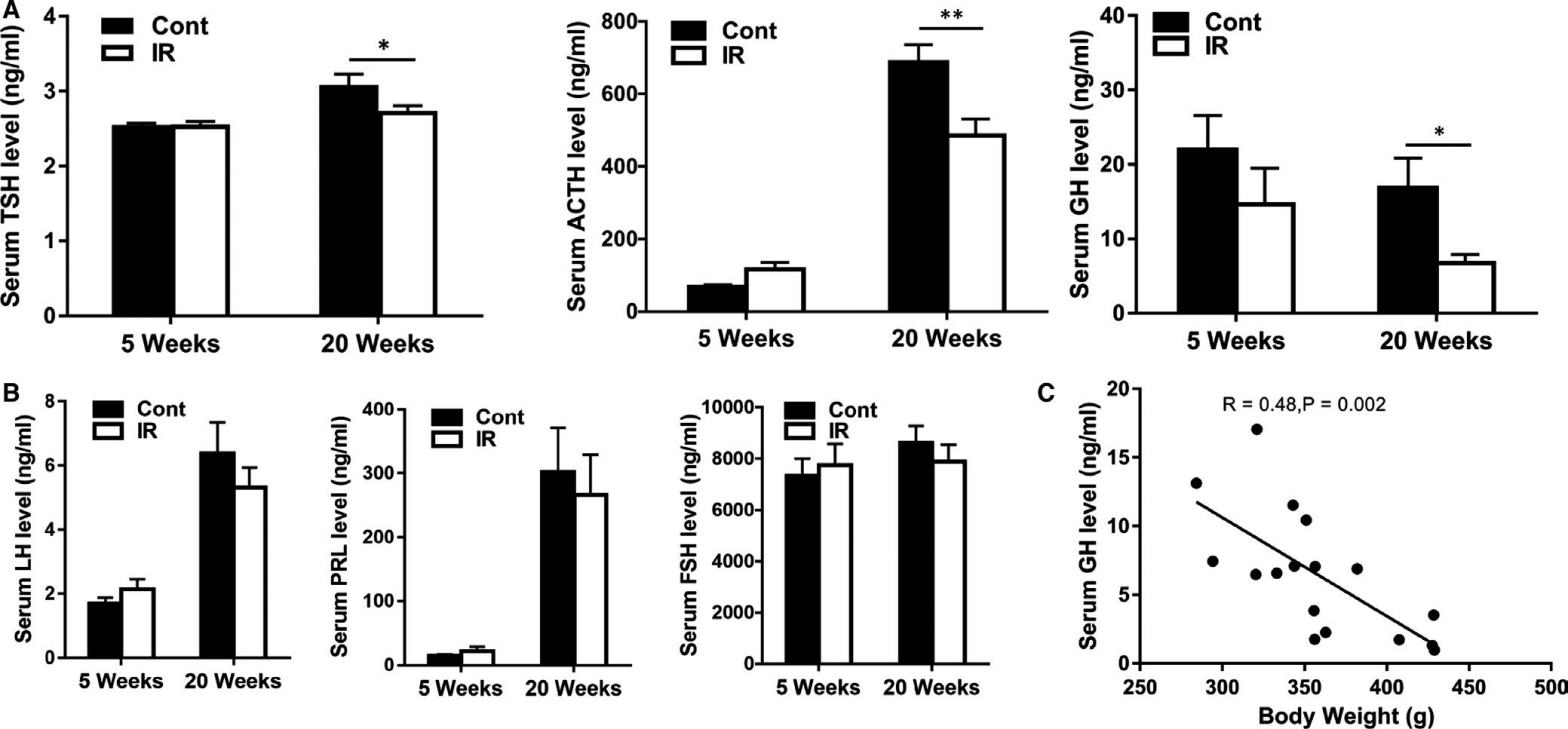
Irradiation‐induced pituitary‐related hormone deficiency. A, The serum ACTH, TSH and GH levels were significantly decreased at 20 wk in the irradiated group compared to the control group, but no difference was seen at 5 wk. B, There was no change in serum LH, PRL and FSH hormone levels after brain irradiation. C, Correlation analysis between serum GH hormone levels and body weight. n = 20/group, *P < 0.05, **P < 0.01

Because the whole transcriptome analysis indicated that endocrine resistance signalling pathways were differently regulated after the irradiation, we further explored whether or not early irradiation might cause endocrine changes in female rats during adulthood. Pituitary‐related hormones, including TSH, ACTH, GH, lutrophin (LH), prolactin (PRL) and follicle stimulating hormone (FSH), were assayed in serum at different time points after irradiation. There was no difference between the irradiated group and the non‐irradiated control group at 5 weeks after irradiation. However, the serum TSH (*P* < 0.5), ACTH (*P* < 0.001) and GH (*P* < 0.05) levels were significantly decreased in the irradiated group at 20 weeks compared to the control group (Figure 6A), but no significant decreases were seen for the serum LH, PRL and FSH levels (Figure 6B). Correlation analysis showed a negative correlation between body weight and serum GH levels (Figure 6C).

### 3.7 Long‐term effects of irradiation on sex hormones and the structures of the reproduction organs

**Figure 7:**
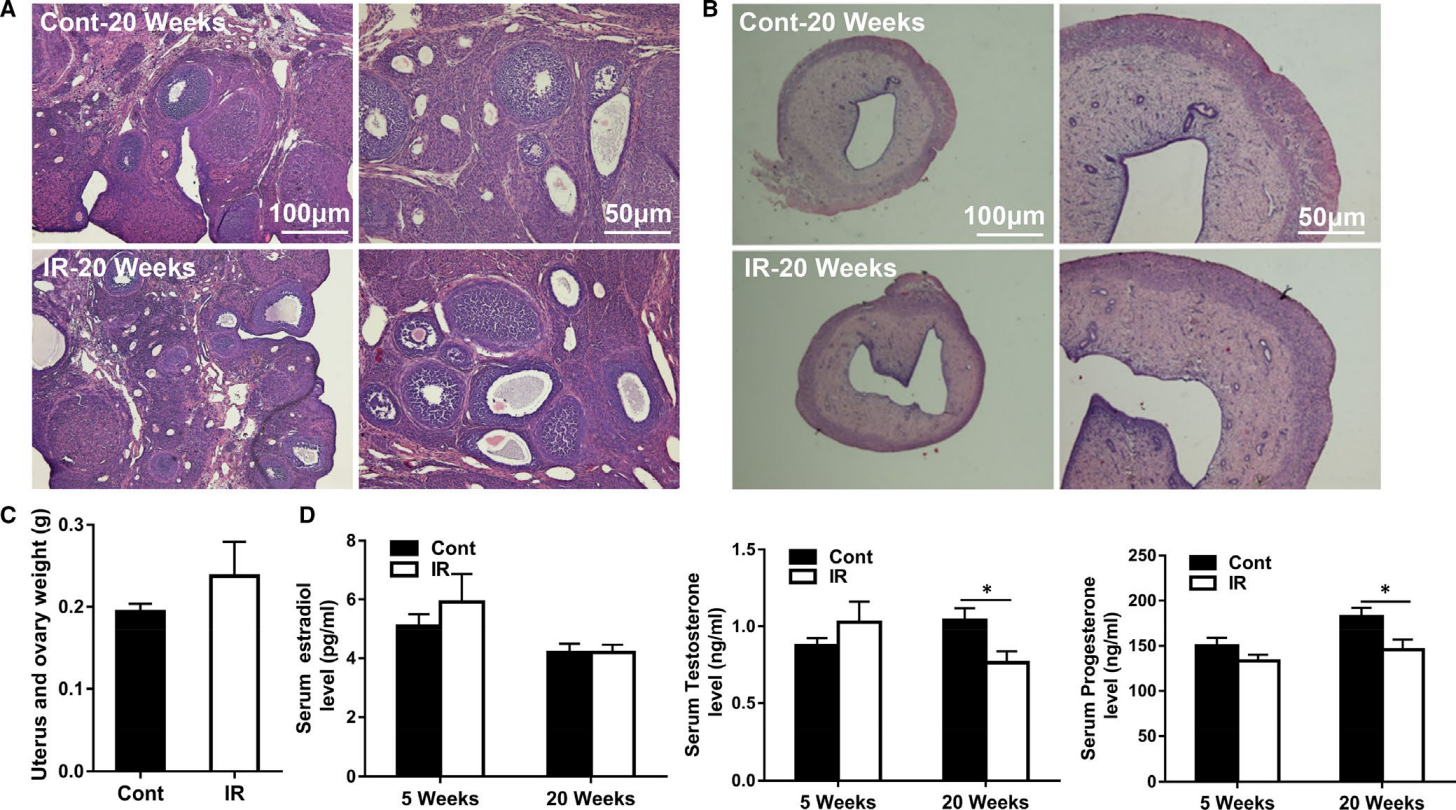
Long‐term effects of irradiation on reproduction. A, Representative H&E staining of the ovary and uterus tissue. B, The tissue weight of the ovary and uterus at 20 wk after irradiation. C, The serum estradiol, testosterone and progesterone levels at 5 and 20 wk. n = 20/group, *P < 0.05

Serum testosterone and progesterone levels were significantly decreased in the irradiated group at 20 weeks compared to the control group during the dioestrus period (*P* < 0.05), but no differences were observed on the serum estradiol level at 5 weeks after irradiation (Figure 7D). Of note, no structural abnormalities were found in the ovary or the uterus at 20 weeks after irradiation (Figure 7A,B), and there were no differences in the weight of the ovary or uterus (Figure 7C) between the irradiation and the control groups.

### 3.8 Long‐term impact of irradiation on the oestrous cycle

The long‐term impact of irradiation on the oestrous cycle was evaluated from 15 weeks after irradiation. The oestrous cycle, as indicated by vaginal smears, was divided into the dioestrus, pro‐oestrus, oestrus and metoestrus phases. Compared with the control group, the irradiation group did not have any abnormalities in oestrous cycle fluctuations (Figure S2A,B), and there were no differences in the durations of each phase (Figure S2C).

## 4 DISCUSSION

The pituitary gland is difficult to keep out of the irradiation field for the treatment of non‐pituitary tumours, including skull base tumours, nasopharyngeal tumours, primary brain tumours, central nervous system prevention of patients with acute lymphoblastic leukaemia, and total body irradiation prior to bone marrow transplantation.^24^, ^25^ Currently, pituitary dysfunction is considered to be the most common complication of CRT, and the largest long‐term study of children treated with chemotherapy and radiotherapy (748 participants treated with CRT with a mean follow‐up time of 27.3 years) showed that the cumulative incidence of GH, TSH, ACTH and gonadotropin deficiency at 40 years of age was 72.4%, 11.6%, 5.2% and 24.4%, respectively.^26^ Another systematic review showed that two‐thirds of patients with head, neck and brain tumours suffer from pituitary dysfunction after CRT.^27^ Of those with pituitary dysfunction, GH deficiency is typically reported to be the first and most common clinical symptom to occur after injury, and this has been confirmed by multiple follow‐up studies.^28^, ^29^, ^30^, ^31^ The risk for pituitary dysfunction increases with the radiation dose delivered to the pituitary axis and the exposure duration, ranging from 25% to 50% after 8 to 14.4 Gy,^32^ 0% to 66% after 18 to 24 Gy^33^ and 80% to 90% after ≥30 Gy CRT.^30^, ^34^ The endocrine complications usually take years to develop, and the clinical symptoms are usually hidden and imperceptible,^35^ but the signalling pathways and the molecular mechanisms, especially in the pituitary after irradiation, remain unknown. Due to the difficulty in using human samples, animal models, especially rat models, have been widely used in pituitary studies. However, the human pituitary is only about 1/3 inch in diameter, and the rodent pituitary is about 10 times smaller, and with such a small organ volume, transcriptome analysis is more advantageous than traditional methods. Our study has for the first time used transcriptomics in an animal model to analyse changes in the pituitary gland after brain irradiation, and we found that the pituitary transcriptome changed significantly after irradiation. Genes usually interact with each other in performing their biological functions, and pathway‐based analysis helps to further understand genes’ biological functions. We performed pathway enrichment analysis of differentially expressed genes (DEGs) based on KEGG (the major public pathway‐related database),^36^ and we identified significant enrichment of endocrine resistance, apoptosis and inflammatory pathways and signal transduction pathways in DEGs compared with the whole‐genome background in the pituitary gland. These results provide direct evidence of injury to the pituitary gland by brain irradiation.

The p53 signalling pathway plays a critical role in safeguarding the integrity of the genome. After the p53 molecule is activated, it initiates cellular processes and exerts most of its tumour‐suppressor functions by binding to the enhancer/promoter element of the downstream target gene and regulating its transcription.^37^ However, excessive activation of the p53 signalling pathway induces either viable cell growth arrest or apoptosis.^38^ In this study, we found that irradiation increased gene expression in the p53 signalling pathway, and this suggests that neuronal apoptosis in the pituitary increases after irradiation. Another established pathway that regulates apoptosis is the Hippo pathway, and this pathway is required for the induction of apoptosis and for the reduction of cell differentiation during development. Unlike the p53 pathway, Hippo pathway‐related gene expression in the pituitary gland decreased significantly after brain irradiation. This suggests that brain irradiation leads to increased apoptosis and decreased cell proliferation by activating the p53 pathway and inhibiting the Hippo pathway, ultimately leading to pituitary injury, and the number of BrdU‐labelled cells in the pituitary was significantly reduced in the irradiated group compared with the non‐irradiated group at 20 weeks, and reduced cell proliferation in the pituitary gland corroborated these RNAseq findings.

Neuroinflammation is a significant problem after irradiation, and our previous studies using the rodent irradiation model to measure inflammation in the cerebellum, hippocampus and hypothalamus showed that irradiation can increase the inflammatory response and the release of inflammatory factors.^22^, ^39^, ^40^, ^41^, ^42^ This neuroinflammation is related to brain damage associated with radiation therapy, and it has been associated with the pathogenesis of various radiation‐induced side effects.^43^, ^44^ In the current study, irradiation increased the expression of pro‐inflammatory factor genes and decreased the expression of anti‐inflammatory factor genes and activated the TNF inflammatory signalling pathway in the pituitary gland, an effect that is similar to other neurogenic regions.^39^, ^44^, ^45^ Also, simultaneous measurement of cytokine/chemokine panels in the pituitary gland using Luminex confirmed that there is an acute inflammatory reaction 6 hours after irradiation and that the chronic inflammatory response lasts for 20 weeks. These results provide a rationale for the use of anti‐inflammatory interventions to ameliorate or prevent radiation‐induced pituitary injury in the juvenile brain.

We also observed a decrease in the serum level of GH, ACTH and TSH after brain irradiation that corroborates the findings from these clinical studies. In addition, we found a negative correlation between body weight and growth hormone levels in rats that also agrees with the notion that GH deficiency leads to obesity, especially increased visceral obesity.^46^ Taken together, our study has confirmed in an animal model the impact of juvenile brain irradiation on pituitary dysfunction in adulthood.

Radiotherapy often results in reduced fertility in adult survivors of childhood cancer. With increasing survival rates, fertility has been recognized as an important life quality concern for cancer patients.^47^ Clinically, CRT might induce infertility by disrupting the hypothalamic‐pituitary‐gonadal axis and by disrupting hormone secretion. Treatment might delay the maturation and normal development of survivors and lead to negative body image and psychological distress.^48^, ^49^ A study of 3,531 female childhood cancer survivors showed that pituitary radiation doses between 1 Gy and 30 Gy significantly increased the risk of infertility.^50^ With the aim to characterize the reproductive changes, we measured the oestrous cycle and the structure of the ovaries and uterus, and these two indicators did not change in female rats after juvenile brain irradiation. However, serum levels of the testosterone and progesterone sex hormones decreased significantly, and this might be due to the fact that low‐dose brain irradiation was used and that rats have more powerful repair and adaptive ability compared to humans. These results suggest that it is worth monitoring serum testosterone and progesterone levels in survivors of female childhood cancer in order to detect early reproductive disturbances.

The pituitary gland is composed of the adenohypophysis and neurohypophysis. The adenohypophysis produces and releases hormones. The neurohypophysis itself does not produce hormones—but this is done by nerve cells in the hypothalamus, which release hormones into the circulation. Hormones in the pituitary gland signal to other endocrine glands to increase or inhibit their own hormone secretion. For example, when one feels stressed, the anterior pituitary lobe releases ACTH, which stimulates the production of adrenal cortisol,^51^ TSH, which stimulates the thyroid to produce hormones, and FSH, which works with LH to ensure the normal function of the ovaries.^52^, ^53^ GH stimulates growth, cell regeneration, and is thus important in human development. Clinical MRI results show that CRT in children has a negative impact on pituitary height, sagittal width and pituitary volume,^54^, ^55^ and the negative effects of irradiation on pituitary function have also been described previously.^8^, ^30^ These results indicate that it is worth monitoring pituitary gland‐related hormones in survivors of childhood cancer in order to detect early endocrine disturbances.

## 5 CONCLUSIONS

This study confirmed that irradiation to the juvenile brain induces cell apoptosis and neuroinflammation in the pituitary gland in the acute phase and reduces pituitary secretion of GH, ACTH and TSH in the chronic phase and thus leads to disorders related to insufficient serum sex hormone levels. Our results provide compelling evidence that radiation‐induced pituitary damage induces a lack of endocrine and reproduction‐related hormone secretion, and cell proliferation and persistent inflammation might play key roles in this process. Importantly, the current study might be used to develop novel strategies for reducing radiotherapy‐induced long‐lasting side effects in the treatment of paediatric brain tumours.

## CONFLICT OF INTEREST

The authors declare that they have no conflict of interest.

## AUTHOR CONTRIBUTIONS

**Yiran Xu:** Conceptualization (equal); Data curation (equal); Project administration (equal); Visualization (equal); Writing‐original draft (equal); Writing‐review & editing (equal). **Yanyan Sun:** Conceptualization (equal); Data curation (equal); Formal analysis (equal); Validation (equal); Visualization (equal). **Kai Zhou:** Conceptualization (equal); Data curation (equal); Methodology (equal); Resources (equal); Writing‐review & editing (equal). **Cuicui Xie:** Data curation (supporting); Formal analysis (supporting); Methodology (supporting). **Tao Li:** Formal analysis (supporting); Funding acquisition (supporting); Investigation (supporting); Methodology (supporting). **Yafeng Wang:** Data curation (supporting); Investigation (supporting); Methodology (supporting); Software (supporting). **Yaodong Zhang:** Data curation (supporting); Formal analysis (supporting); Investigation (supporting); Methodology (supporting). **Juan Rodriguez:** Data curation (supporting); Formal analysis (supporting); Methodology (supporting). **Xiaonan Zhang:** Funding acquisition (supporting); Methodology (supporting); Project administration (supporting). **Ruijin Shao:** Data curation (supporting); Formal analysis (supporting); Methodology (supporting). **Xiaoyang Wang:** Conceptualization (supporting); Data curation (supporting); Visualization (supporting); Writing‐review & editing (supporting). **Changlian Zhu:** Conceptualization (lead); Data curation (lead); Funding acquisition (lead); Project administration (lead); Resources (lead); Writing‐review & editing (lead).

## Supporting information

Fig S1

Fig S2

## Data Availability

The transcriptome sequencing data generated in this study have been deposited in NCBI BioSampledatabase (https://www.ncbi.nlm.nih.gov/bioproject/PRJNA528821), and the data are available from the corresponding author on reasonable request.
