# Supplementary figures and images for: Cranial irradiation alters neuroinflammation and neural proliferation in the pituitary gland and induces late‐onset hormone deficiency
Source: J Cell Mol Med. 2020 Nov 10;24(24):14571–82. doi: 10.1111/jcmm.16086 (PMC7754041; doi:10.1111/jcmm.16086)

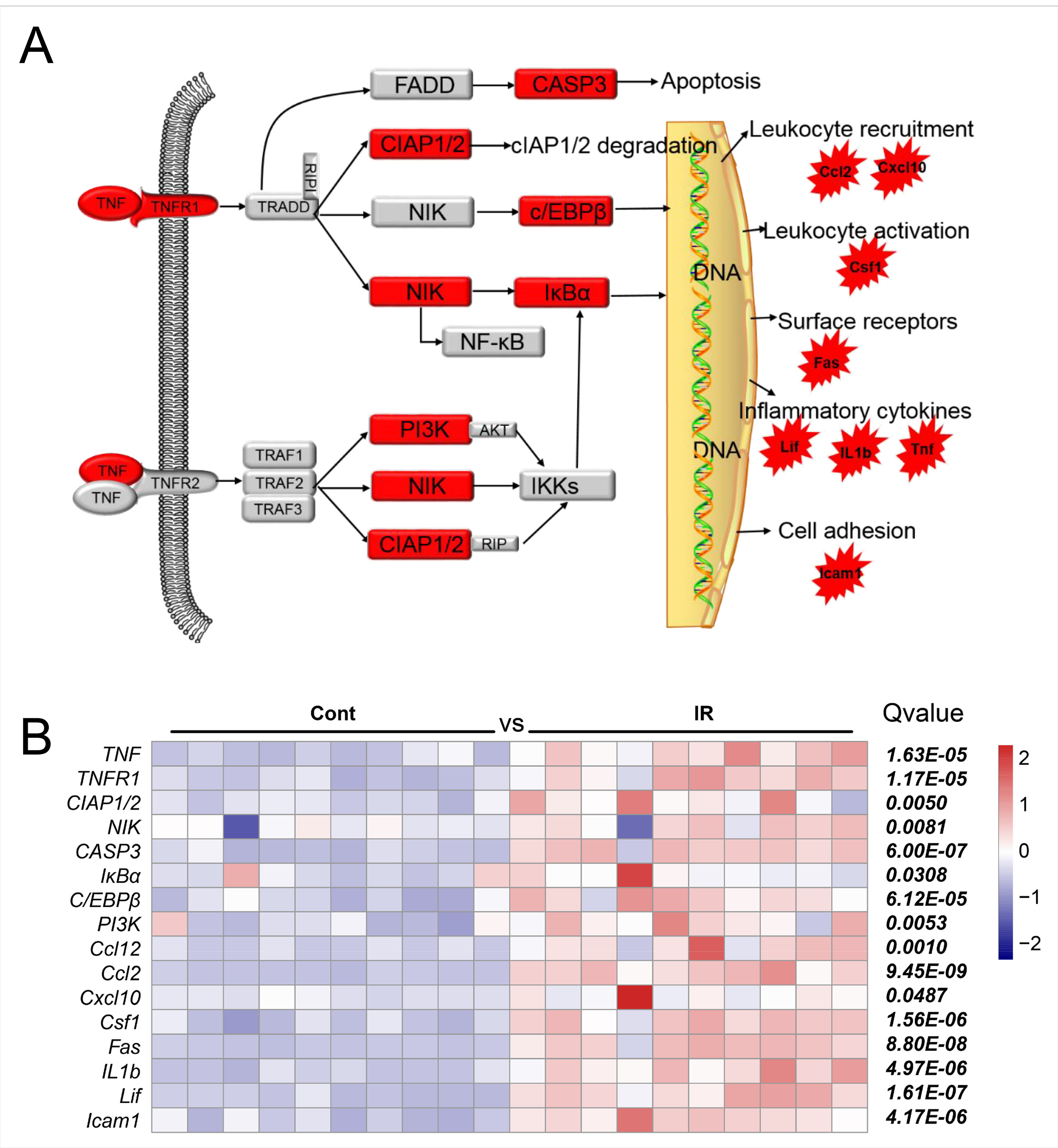

Supplement: Supplementary file 1 — Fig S1 [file JCMM-24-14571-s001.tif]

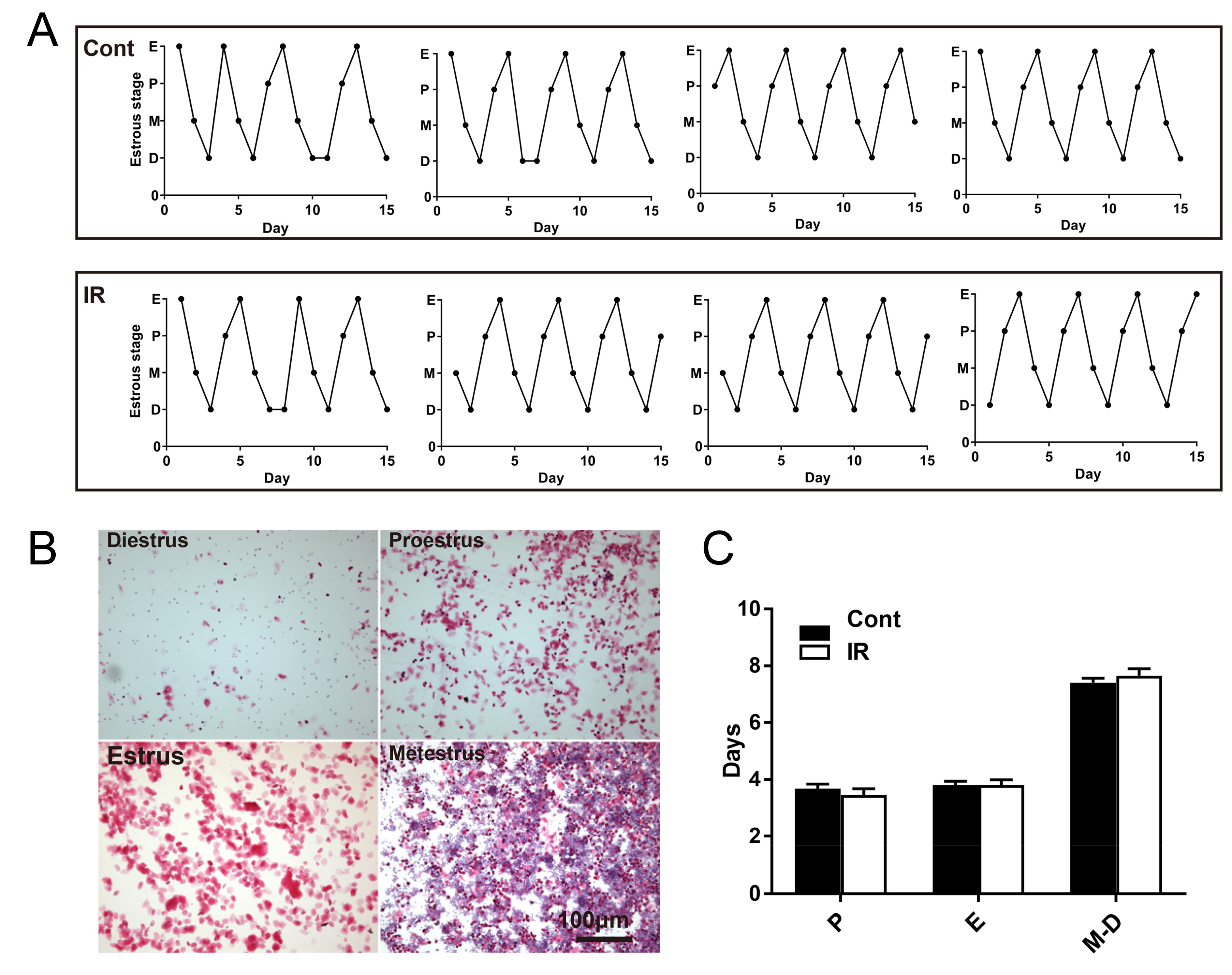

Supplement: Supplementary file 2 — Fig S2 [file JCMM-24-14571-s002.tif]
